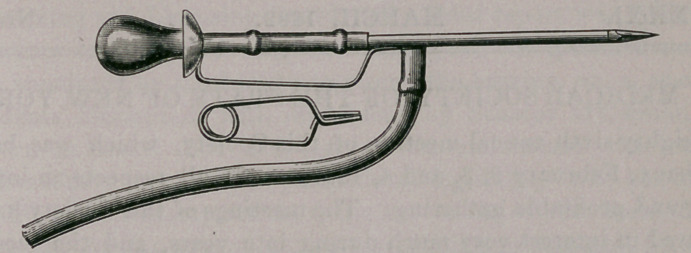# A Pleura-Trocar

**Published:** 1892-03

**Authors:** Marcell Hartwig

**Affiliations:** Buffalo, N. Y.


### A PLEURA-TROCAR.

By MARCELL HARTWIG, M. D., Buffalo, N. Y.

THIS instrument, which I have recommended and described in the *Medical Record* of January 17, 1891, page 80, has served me practically for years. I have been satisfied with it every time, so that I can heartily recommend it to the profession. It admits perfect antiseptising (boiling), and excludes the air. A pump is not needed. About four feet of rubber tubing will syphon the pleural cavity perfectly.

Oil the stylet with an antiseptic vaseline, and compress the rubber interstice gently with two fingers while withdrawing the stylet. Keep up the compression with the hand holding the trocar; a light clamp can substitute the fingers. At a time when the diagnosis of pleural exudation becomes possible, there is probably always pressure enough in the pleural cavity to expel the air from the syphoning tube.

For safety's sake, though, the tube should be filled with an antiseptic fluid, when the trocar is plunged into the intercostal space in order to start the syphoning, while the end of the tube dips into the same fluid in a dish on the floor, and always test the perfect fitting of the rubbers with water before beginning an operation. Of course, injection can be made through the same tube.

Stoddard Bros., of Buffalo, have constructed the trocar to my entire satisfaction.

---

HE WAS NO FLY-ROOST.—Shingler—" I have called to ask your daughter's hand in marriage."

Prospective Pa—" But you are an unknown doctor, without sufficient income to support her, and the ethics of your profession forbid you to advertise."

Shingler—" Yet I am no fly-roost. I have let three rooms over my office to reporters, have given them free use of my telephone, and have joined the Press Club."—*Munsey's Weekly*.

## Figures and Tables

**Figure f1:**